# Supplementary material for: Perioperative Management and Outcomes after Endovascular Mechanical Thrombectomy in Patients with Submassive (Intermediate-Risk) Pulmonary Embolism: A Retrospective Observational Cohort Study
Source: Healthcare (Basel). 2024 Aug 27;12(17):1714. doi: 10.3390/healthcare12171714 (PMC11395241; doi:10.3390/healthcare12171714)
Supplement: Supplementary file 1 [file healthcare-12-01714-s001.zip › healthcare-3124737-supplementary.pdf]

Supplemental data 1

The first patient that died was a 61-year-old female with BMI 41.2 that presented after three days of generalized weakness and lightheadedness that suffered a syncopal event. She was found to be hypoxemic with oxygen saturation of 85% on room air, heart rate of 131 and blood pressure of 107/71. She had a past medical history of hyperlipidemia, obesity, and degenerative low back pain. She had no history of prior PE or DVT, active cancer, recent surgery, and no contraindication to thrombolytics. A CTAP revealed extensive pulmonary embolic disease to include a saddle PE with extension into the left main and the entire left pulmonary artery system and right main pulmonary artery and had paradoxical bowing of the interventricular septum with RV strain. Laboratory evaluation revealed both elevated cTnT level of 114 ng/L and BNP of 2974 pg/mL. The calculated PESI score was 231 making this a submassive and intermediate-high risk PE. The patient was started on IV Heparin and the pulmonary embolism response team (PERT) was consulted and recommended MT which was performed within 2.5 hours of initial evaluation in the emergency department. The interventional radiologist was able to advance the 16 French Flash penumbra aspiration catheter into the left main pulmonary artery revealing a mean PAP of 46. Two passes were unsuccessful in aspirating any thrombus and then the patient's oxygen saturation started to decline into the 70's and became unresponsive with a code blue being called. The patient was promptly intubated and then went into a PEA cardiac arrest and CPR was initiated. Following five rounds of Epinephrine and CPR, the decision was made to proceed with 50 mg bolus of Alteplase. However, after another 15 minutes of CPR the patient's TTE continued to demonstrate severely dilated RV and no cardiac activity and no pericardial effusion. At this time, it was felt that there was no further intervention, and the resuscitation efforts were terminated.

The second patient that died was a 73-year-old female with a BMI of 26.5 that presented with three days of chest pain and dyspnea on exertion to the emergency department. She has a history of active metastatic colon cancer involving pancreas, liver, colon, peritoneal carcinomatosis, intra-abdominal lymphadenopathy and bilateral lungs that had started on chemotherapy two week prior to presentation. Her initial oxygen saturation was 90% on room air, heart rate of 128 and blood pressure of 88/64 but improved to 102/68 on the second blood pressure reading. CTAP revealed significant PE burden with saddle embolus as well as extension of the embolus into the right and left main pulmonary arteries and into the lobar and segmental branches of all the lobes and no signs of RV strain. TTE also failed to demonstrate any RV strain with normal RV size and a RV systolic pressure of 42 mmHg. She had no prior history of PE or DVT but was found to have an acute DVT. Laboratory evaluation revealed both an elevated cTnT level of 32 ng/L and BNP level of 1,047 pg/mL. The calculated PESI score was 153 making this a submassive and intermediate-low risk PE. The patient was started on IV Heparin and interventional radiology was consulted and recommended continuing IV Heparin with close observation for deterioration. The next day the patient had continued tachycardia in the 120-130's with repeat TTE now demonstrating moderate RV dilation with RV strain and remained hemodynamically stable so underwent MT and inferior vena cava filter placement for submassive and intermediate-high risk PE. The MT was successful in removing a large amount of thrombus from both right and left pulmonary arteries with pre-procedure mean PAP of 19. Immediately following the procedure, the patient's heart rate decreased to 112 beats per minute and was weaned off oxygen. Follow up TTE the next day after the procedure demonstrated continued moderately dilated RV with a decrease in RV systolic pressure to 34 mmHg from 42 mmHg. The patient's hospital course was complicated by acute kidney injury from pre-renal etiology, constipation and melanotic stools but was able to continue IV Heparin. She was subsequently transitioned to Apixaban five days after the procedure and then discharged to a skilled nursing facility on post procedure day seven due to continued generalized weakness and lower extremity edema. Patient subsequently died ten days later of her cancer progression without any further details available.
